# From tackles to trauma: investigating the influence of the FIFA World Cup 2022 on public maxillofacial department in Qatar - an observational study

**DOI:** 10.1097/JS9.0000000000000804

**Published:** 2023-10-04

**Authors:** Afaf K. Hamze, Abdelfatteh El Omri, Ahmed N. Derbas, Ismail Dergaa, Gustavo Grimaldi Finol, Noomen Guelmami, Mohamed Amine Rejeb, Antonio J. Santimano, Kalpana Singh, Omar M. Aboumarzouk, Moustafa Al Khalil

**Affiliations:** a Oral and Maxillofacial Surgery; b Surgical Research Section, Department of Surgery; c Nursing Research, Hamad Medical Corporation; d Primary Health Care Corporation (PHCC); e Clinical and Applied Health Research Department, QU-Health, College of Medicine, Qatar University, Doha, Qatar; f High Institute of Sport and Physical Education, University of Sfax, Sfax, Tunisia; g Postgraduate School of Public Health, Department of Health Sciences (DISSAL), University of Genoa, Genoa, Italy; h Department of Surgery, School of Medicine, Dentistry and Nursing, The University of Glasgow, United Kingdom of Great Britain and Northern Ireland, Glasgow, United Kingdom

The Federation Internationale de Football Association (FIFA) World Cup (FIFA-WC) is considered the pinnacle of international sporting events, attracting a diverse array of attendees from across the globe^1^. Hosting such an event presents diverse challenges for the organizing country, which are further amplified by public health threats^1^. The 22nd FIFA-WC, held in Qatar from 20 November to 18 December 2022, distinguished itself in several unprecedented dimensions. It marked the first instance of a FIFA-WC being conducted amidst the throes of a global pandemic, specifically the COVID-19 crisis^1,2^. Moreover, Qatar’s demographic and geographic attributes further accentuated its unique position as the smallest nation, both in terms of population and territory, to host such a monumental event^1,2^. Notably, the event was devoid of the previously mandated COVID-19 restrictions, as they were lifted in September 2022, presenting an unparalleled scenario for the healthcare sector.

Historically, large-scale sporting events like the FIFA-WC have been associated with a decrease in routine healthcare activities. This pattern has been ascribed to a multitude of factors, including the appeal of live matches, large fan congregations obstructing access to healthcare facilities, and apprehension of violence, among others^3^. Indeed, according to Zrobak *et al*.^3^, Cape Town witnessed fewer emergency department visits for traumatic injury during the 2010 FIFA-WC in South Africa. They further reported a decrease in all-cause pediatric emergency department visits during hometown matches^3^. Similarly, a study conducted by Alesandrini *et al*.^4^ in Nancy, France, revealed significant disparities in Pediatric Emergency Department attendance during the 2018 FIFA-WC and the 2016 UEFA championships^4^.

The primary objective of this study was to elucidate the ramifications of the FIFA-WC 2022 on a specific specialty clinic in Qatar, focusing on the national maxillofacial surgery department. The emphasis was placed on a comparative analysis of surgical activities during the tournament vis-à-vis the analogous period in the preceding year. This study aimed to shed light on how such a significant international event impacts public health activities, particularly in specialized fields like maxillofacial surgery, amidst the COVID-19 pandemic. Contrary to the anticipated decrease in healthcare activities, the FIFA-WC 2022 in Qatar witnessed a marked surge in maxillofacial surgeries relative to the corresponding timeframe in 2021. During the period before the start of the FIFA World Cup Qatar 2022 period (pre-WC), there was a highly significant increase in the number of surgeries conducted in 2022 compared to 2021 (*P*<0.0001, *χ*^2^=176 989). In contrast, the period during the FIFA-WC 2022 (WC) itself did not show a significant difference in the number of surgeries between 2022 and 2021 (*P*=0.088, *χ*^2^=2909). However, the period after the FIFA-WC2022 (post-WC) demonstrated a significant increase in the number of surgeries conducted in 2022 compared to 2021 (*P*<0.001, *χ*^2^=10.1). When considering the total study period, including all the selected time frames (Total), there was a highly significant increase in the number of surgeries conducted in 2022 (507 episodes) compared to 2021 (343 episodes) (*P*<0.0001, *χ*^2^=36.09), predominantly male patients (90.3%) with an average age 33.4±13.6. Analyzing the group stage of the FIFA-WC2022 (WC-S1), it was found that there was a highly significant increase in the number of surgeries conducted in 2022 compared to 2021 (*P*<0.0001, *χ*^2^=30.13). On the other hand, during the period between the Round 16 and the final game of the FIFA-WC2022 (WC-S2), there was no significant difference in the number of surgeries between 2022 and 2021 (*P*=0.06, *χ*^2^=3.54). In summary, all results of the conducted surgeries are visually represented in Figure 1. For a detailed comparison between the maxillofacial surgeries conducted in 2021 and 2022, readers are referred to Supplementary Table 1 (Supplemental Digital Content 1, http://links.lww.com/JS9/B167). Additionally, a comprehensive breakdown of the types of surgeries is provided in Supplementary Table 2 (Supplemental Digital Content 2, http://links.lww.com/JS9/B168).

**Figure 1:**
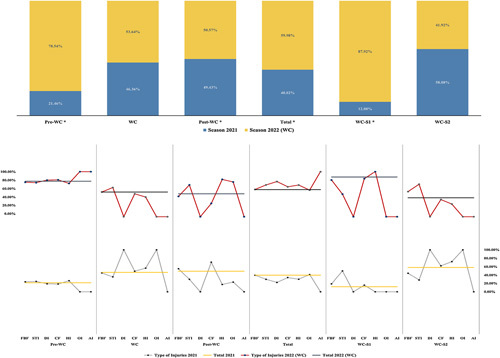
Trends in maxillofacial surgeries during the FIFA World Cup 2022 in Qatar: a comparison between 2021 versus 2022. *Data were % and mean ± SD for categorical and quantitative data*. *: (P<0.001: Significant difference between season 2021 and 2022). *FIFA-WC2022:* FIFA World Cup Qatar 2022; *Pre-WC:* The period before the start of the FIFA World Cup Qatar 2022 (FIFA-WC2022) (1 November–19 November 2022); *WC:* The period during the FIFA-WC2022 (20 November–18 December 2022); *Post-WC:* The period after the FIFA-WC2022 (18 December–31 December 2022); *Total:* The entire FIFA-WC2022 period (1 November–31 December 2022); *WC-S1:* The group stage of the FIFA-WC2022 (20 November–2 December 2022); *WC-S2:* The period between the Round 16 and the final game of the FIFA-WC2022 (3 December–18 December 2022); *FBF:* facial bones fractures; *STI:* soft tissue injuries; *DI:* dental injuries or alveolar bone fracture; *CF:* complex fracture; *HI:* head injuries; *OI:* orthopedic injuries; *AI:* abdominal injuries.

Initially, one might conjecture that this increase in maxillofacial surgeries could be indicative of a surge in violent incidents or hooliganism^5^, as has been observed in previous World Cups^3,6–8^. However, the FIFA-WC 2022 in Qatar diverged remarkably from previous World Cup experiences. It was reported to be one of the safest World Cups ever held^8^. Indeed, the data reveals a striking absence of causes related to match violence among the admissions to the trauma center. While the specific reasons for admission to the trauma center were not available in the dataset – a limitation of this study – it is noteworthy that none of the admissions were related to FIFA-related mass gatherings.

This absence of violence-related admissions, despite the significant increase in surgeries, compelled us to reconsider the underlying factors contributing to this trend. It effectively negated the hypothesis that the uptick in surgical procedures was a consequence of hooliganism or violence typically associated with such large-scale events.

Accordingly, we posited alternative hypotheses to account for this unexpected pattern. One such hypothesis is that the strategic and proactive healthcare planning by Qatari authorities, coupled with the introduction of telemedicine consultations for non-emergent cases^2,9^, may have enabled more efficient utilization of healthcare resources. This, in turn, could have allowed for the accommodation of a greater number of surgical procedures unrelated to the World Cup events.

Furthermore, Qatar’s prior experience in orchestrating events during the pandemic, such as the Emiri Cup, the AFC (Asian Football Confederation), and notably the Arab Cup^1^, may have provided invaluable insights and training for healthcare staff, policymakers, police, traffic department, and infection control specialists. This long-term vision and experience likely contributed to the successful management of the FIFA-WC 2022 event under challenging circumstances.

Another hypothesis could be related to Qatar’s robust infrastructure and efficacious traffic management, which ensured unhindered access to healthcare facilities. This was particularly salient given the influx of ~1.7 million fans, a significant proportion relative to Qatar’s indigenous population^1^. Despite this influx, the healthcare system demonstrated the capacity to handle the increased density of road traffic and ensure timely access to healthcare facilities.

In conclusion, this study has revealed unexpected findings regarding the impact of the FIFA-WC 2022 on the national maxillofacial surgery department in Qatar. Contrary to expectations and historical trends, the number of surgeries during the tournament period in 2022 was higher compared to the previous year. This increase can be attributed to effective strategic planning and coordinated healthcare services by the authorities, which contributed to the functioning of the healthcare system during the event. The findings of this study have practical implications for future event planning, highlighting the importance of meticulous coordination and resource allocation. The lessons learned from Qatar’s experience can provide valuable insights for other countries hosting major sporting events. By adopting proactive approaches, countries can better prepare their healthcare systems to address the challenges associated with hosting global sporting events. This study contributes to improved preparedness for future events and provides valuable insights into maintaining healthcare services and enhancing patient care during mass gathering events.

## Ethical approval

Exempted IRB ethical approval and approved by HMC-MRC surgical research section under the reference SR/RE/2022/42.

## Consent

Not applicable.

## Sources of funding

The study did not receive any funding.

## Author contribution

A.K.H., A.E.O., M.A.K., A.N.D., and G.A.G.F.: conception and design; M.A.R. and A.J.S.: data collection and trimming; A.E.O., O.M.A., and A.K.H.: data quality verification; N.G., K.S., I.D., and A.E.O.: analysis and interpretation of the data; I.D. and A.E.O.: drafting of the paper; A.K.H., O.M.A., M.A.K., and A.E.O.: revising it critically for intellectual content. All authors gave their final approval to the version that will be published.

## Conflicts of interest disclosure

The authors declare that the research was conducted in the absence of any commercial or financial relationships that could be construed as a potential conflict of interest.

## Research registration unique identifying number (UIN)

Not applicable.

## Guarantor

The corresponding author, Dr Abdelfatteh El Omri, PhD.

## Data availability statement

The data that support the findings of this study are openly available upon reasonable request from the corresponding author.

## Provenance and peer review

Not commissioned, externally peer-reviewed.
